# Can Metagenomic Analyses Be Used Effectively in Safe Food Production?

**DOI:** 10.1002/fsn3.70772

**Published:** 2025-08-07

**Authors:** Berrak Delikanli‐Kiyak, Ilkay Yilmaz, Metın Guldas

**Affiliations:** ^1^ Food Processing Program, Vocational School of Iznik Bursa Uludag University Ni̇lüfer/Bursa Turkey; ^2^ Department of Gastronomy and Culinary Arts, Faculty of Fine Arts, Design and Architecture Baskent University Etimesgut/Ankara Turkey; ^3^ Department of Nutrition and Dietetics, Faculty of Health Sciences Bursa Uludag University Bursa Turkey

**Keywords:** food safety, foodborne pathogens, metagenomic analysis

## Abstract

This review aims to bring together current research on the importance, recent applications, and possible impacts of metagenomic analysis and emphasize that metagenomic analyses, which provide direct analysis of the genomes of the food itself and all the microbiota in its environment, are revolutionary approach for effective management of food safety requirements. The increasing health problems in recent years and the related developments regarding nutritional awareness have led people to seek safe food consumption. In order to ensure the quality and safety of food products and protect the health consumers worldwide, necessary preventive measure should be taken by conducting innovative control methods. Numerous microbial hazards threaten food safety worldwide. In this respect, rapid and accurate detection of pathogens is critical. Although traditional culture‐based methods continue to be widely used, they often fail to detect organisms that are viable but cannot be cultured. This limitation has led to the increasing adoption of metagenomic analyses, which allow direct genome‐level detection and provide a more comprehensive view of microbial communities in food systems. In this context, metagenomic analyses within new‐generation technology has been inspired, as it can be a powerful tool to ensure food safety. In this study, for this purpose, the keywords ‘metagenomics and food’ and the studies on metagenomics published in English, peer‐reviewed journals, and available in the Web of Science and Scopus databases were investigated. The years 2020–2024 were taken as a basis for the studies with the keywords identified and studies on antibiotic resistance and unrelated foods were excluded from the review's scope. This review brings together current research on the significance of metagenomics analysis in food safety, its latest applications, and possible effects. The study is a reference source for potential studies to be carried out in this context.

## Graphical abstract

This graphical abstract illustrates the metagenomic workflow applied in food safety. It outlines the process from microbial detection in food matrices to genome‐level identification via next‐generation sequencing (NGS). The visualization emphasizes the integration of modern sequencing technologies to assess foodborne pathogens and microbial communities more accurately and rapidly, ensuring enhanced food safety monitoring and control.

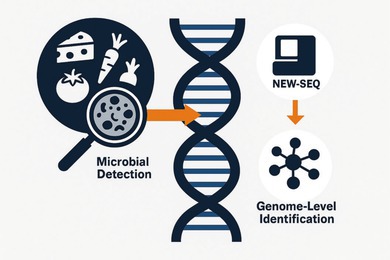

## 1 Introduction

**FIGURE 1:**
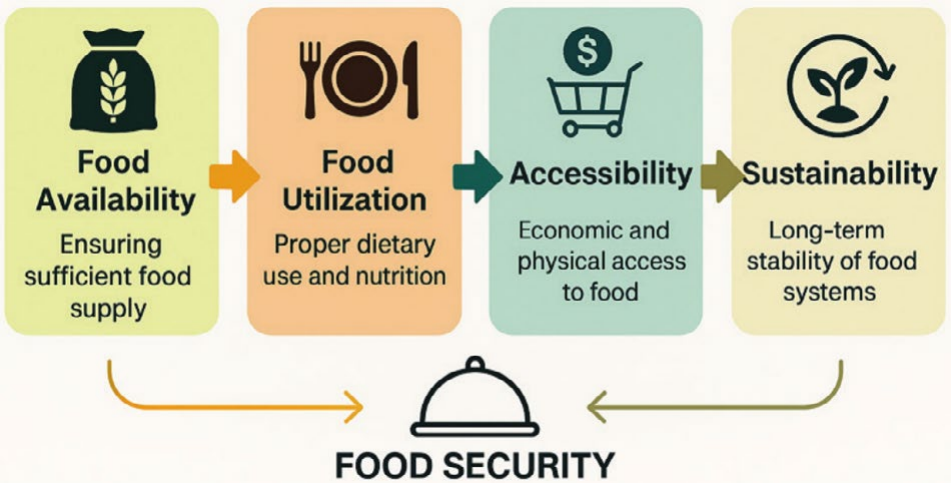
Key drivers underpinning the global demand for food security.

Food safety is defined as the process that includes all precautions to be taken to foresee and eliminate any physical, chemical, or biological hazards that may occur in foods to protect human health throughout all steps from the production of foods to their consumption, also referred to as the principle of farm to fork or field to table (Awulachew 2024). It has been stated that food safety or food security consists of four basic elements (Figure 1); the ability of consumers to find, access, and benefit from the basic foods they need to live, and the ability to maintain this situation in a stable manner (Koç and Uzmay 2023; Shabir‐Ahmad et al. 2023). Moreover, it is also emphasized that food security is a concept that can change depending on many factors such as time, person, country, etc., and that the boundaries of food safety cannot be clearly drawn due to the multitude of variables in food accessibility.

**FIGURE 2:**
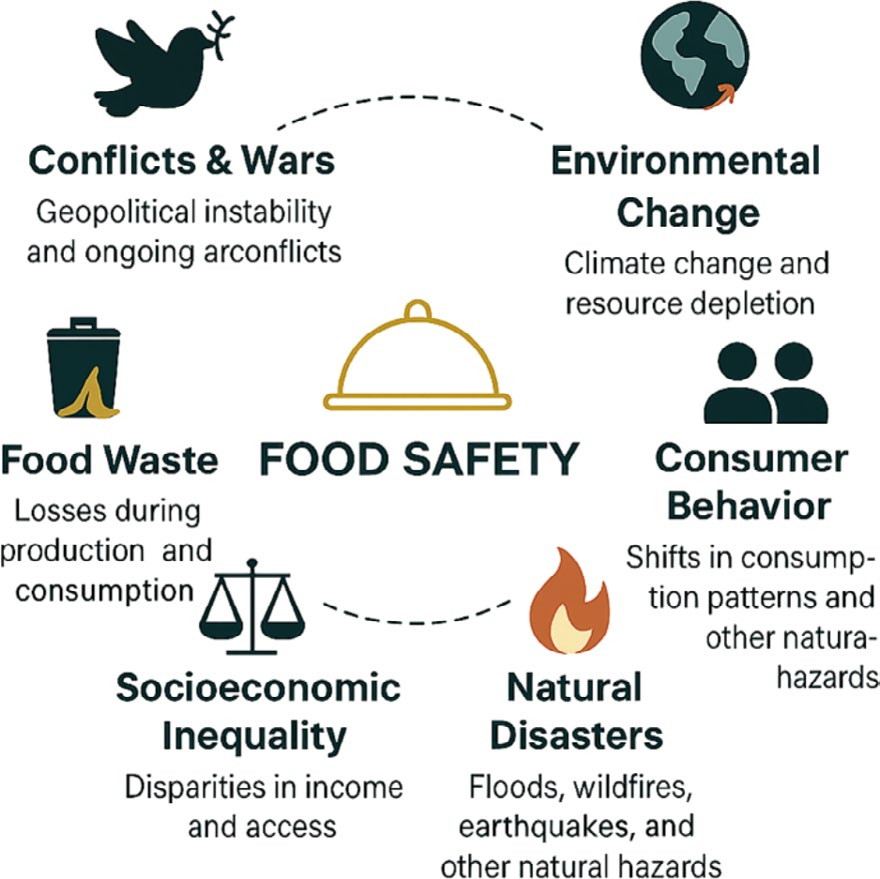
Global determinants threatening food safety.

When the studies completed in this field to date are reviewed and viewed from a historical perspective, food security has been needed due to many global problems (Figure 2). However, increasing food‐based health and nutrition problems in recent years have given serious impetus to the development of innovative methods to ensure food safety. For this reason, food safety is not a problem for yesterday or today only; it should also be considered one of the biggest problems in the future. To support the conceptual flow and improve clarity in visual representation, Figures 1 through 5 were prepared using AI‐assisted illustration tools. These figures were fully designed and verified by the authors to ensure scientific accuracy.

Today, food safety is considered one of the greatest global concerns due to the potential health problems that may arise from foods contaminated by pathogenic microorganisms (Vågsholm et al. 2020; Mainardi and Bidoia 2024). Despite the increase in public awareness and advances in food technology, microbial hazards and infections in food have caused more than 548 million illnesses and 230,000 deaths annually worldwide in the last decade (World Health Organization 2015; Billington et al. 2022). The possibility of serious cases arising from a negligence, delay, or deficiency in the food safety chain should be considered a threat to the health of the individual and society. In order to eliminate the threat, food safety must be fully ensured, controls must be carried out in this direction, deficiencies must be detected, and necessary preventive actions must be implemented depending on the findings obtained. In this context, in order to manage risks in the area of food technology, pathogens in a food sample must be detected and defined in a fast and accurate way (Mainardi and Bidoia 2024). At this point, it is of great importance to implement food safety tests effectively, precisely, and quickly, which have become indispensable for the supply chain by ensuring that food is safe for human consumption. Food safety tests are considered a scientific basis for evaluating the microbiological, physical, or chemical composition of a food product and ensuring that this product is safe for human consumption. In this context, the growth of the food safety testing market and the development of innovative approaches are inevitable. From this point of view, microbiological analyses are considered a critical stage and are classified into two categories: traditional and modern (Kamboj et al. 2020; Gallo et al. 2020; Pampoukis et al. 2022; Sornoza 2024). Traditional methods include culture‐based traditional techniques performed using selective media, which are reliable. However, it is also a known fact that traditional microbiological analyses in studies conducted on food safety have significant problems in determining exactly what the threat to food safety is quickly and effectively. In this regard, their highly time‐consuming nature limits their detection preferences (Kamboj et al. 2020; Gallo et al. 2020). On the other hand, modern methods provide rapid identification, are extremely sensitive, and provide information about microbial diversity and potential contamination sources, allowing them to detect even low‐level pathogens (Pampoukis et al. 2022; Sornoza 2024). In this context, metagenomic analyses are defined as new‐generation technology to provide more accurate analysis results related to food safety (Table 1). Metagenomic analysis examines genetic material obtained directly from environmental or food‐related samples using techniques such as 16S rRNA amplicon sequencing for taxonomic profiling and shotgun sequencing for functional and high‐resolution analysis. Moreover, metagenomic analyses are revolutionary in food safety testing, enabling the direct determination of the genomes in the foods and all microbiota in their environment, as well as measurement of the microbial population (Billington et al. 2022; Sadurski et al. 2024; Muñoz‐Martinez et al. 2025).

**TABLE 1:** The purposes of metagenomic analyses developed in recent years.

Research criteria	Purpose	References
Detection of Foodborne Pathogens	Detection of pathogenic microorganisms avalaible in food production (raw materials, equipment, or production environments) Evaluation of microbial risks (bacterial remaining on food production surfaces) Determination of appropriate guidelines for food safety	(D'aes et al. 2022; Kahraman‐Ilıkkan and Bağdat 2022; Indio et al. 2024; Delikanlı‐Kiyak and Yılmaz 2024)
Monitoring Microbial Diversity	Presence and diversity of various microorganisms by analyzing the genetic structure of microbial flora in foods Determination of the microbial contamination risks	(Das and Tamang 2023; Cerit et al. 2024; Kawooya et al. 2024; Kodape et al. 2024)
Probiotics	Evaluation of probiotic content	(Yasir et al. 2022; Chen et al. 2024)
Process Monitoring and Control	Monitoring and controlling of microbial parameters during the food production processes (e.g., fermentation)	(Cerit et al. 2024; Chen et al. 2024; Guzmán et al. 2020; González‐Orozco et al. 2023; Rimbawan et al. 2024; Slamandane et al. 2024)
Quality Assurance	Evaluation of microbial stability Determination of the microbial quality in the food products Shelf‐life studies	(Luo et al. 2024)
Detection of Microbial Origin	Studies on geogrophical indication	(Slamandane et al. 2024; Emamjomeh et al. 2023; Rustemoğlu et al. 2023)

Traditional culture‐based microbiological methods have been the cornerstone of food safety testing for decades. However, these methods are limited in several key respects. First, they are time‐consuming, often requiring 24–72 h or more for microbial growth and identification. Second, they typically detect only a subset of viable and culturable microorganisms, thus missing a large proportion of viable but nonculturable (VBNC) or fastidious organisms. Third, culture‐based techniques often lack the taxonomic resolution to distinguish closely related species or strains critical for outbreak investigations. In contrast, sequencing‐based metagenomic approaches can detect the entire microbial community, including VBNC and non‐culturable organisms, with high taxonomic precision within hours. Moreover, shotgun metagenomics enables simultaneous functional profiling of genes related to virulence, resistance, or degradation that culture‐based assays cannot access. Although the upfront costs of metagenomics are higher, it significantly reduces the time to results and increases detection accuracy, making it a powerful tool in modern food safety systems (Sadurski et al. 2024; Buytaers et al. 2024; Quek et al. 2025).

**FIGURE 3:**
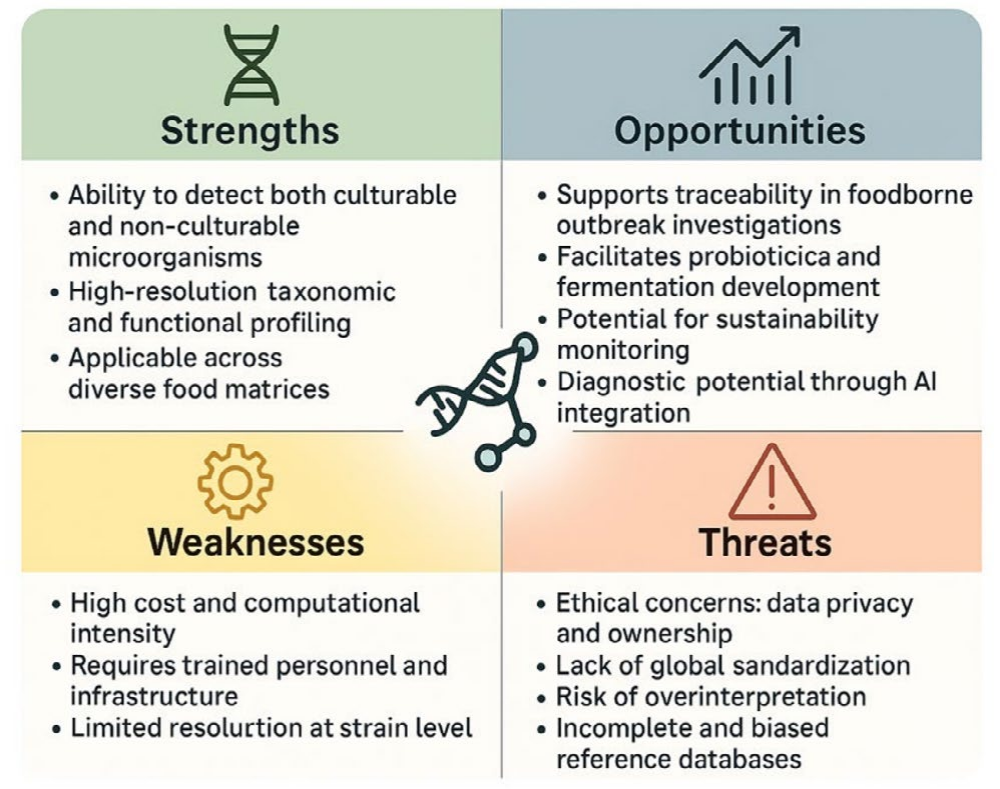
SWOT analysis of the metagenomic analysis approach.

Although the concept of metagenomics was first introduced by Handelsman et al. (1998), its development in the food industry and use as a potential source for understanding microbial diversity, functionality and interactions have become widespread in the last decade (Laudadio et al. 2019; Upadhyay et al. 2023). However, as with all new approaches, innovative metagenomic analyses have revealed some risks and challenges in addition to their advantages when used routinely (Figure 3) (Billington et al. 2022; Brandt et al. 2020; Kim et al. 2022; Sadurski et al. 2024; Sandås et al. 2024). These risks and challenges have also brought some hesitation and regulatory debate, particularly regarding standardization of metagenomic workflows, interpretation of complex data outputs, and validation of results for decision‐making in food safety protocols (Kumar et al. 2017; Niya et al. 2024). Despite these limitations, the prospects for metagenomics approaches are promising. As the food industry continues to develop, it is expected that continued advances in microbiological analysis will play an important role in securing the food supply and maintaining consumer confidence (Sornoza 2024). In this context, the continuous development of new sequencing technologies and bioinformatics tools will significantly increase the sensitivity, accuracy and speed of metagenomics analyses (Niya et al. 2024; Zhang et al. 2019).

Despite the promising applications of metagenomic analyses in food safety, practical implementation still faces significant challenges. One of the most cited barriers is the high cost of next‐generation sequencing (NGS) technologies. However, recent advancements such as portable sequencing devices (e.g., Oxford Nanopore MinION) and cost‐sharing platforms in public health laboratories have made it increasingly feasible for routine use. Another major hurdle is the complexity of bioinformatic data analysis, which requires specialized computational skills. To address this, several user‐friendly pipelines (e.g., QIIME2, MG‐RAST) and cloud‐based analysis tools have been developed, reducing the technical threshold for implementation in nonacademic food laboratories. Additionally, the standardization of protocols and databases, as suggested by the European Food Safety Authority (EFSA), continues to enhance the reliability and reproducibility of metagenomic workflows in the food industry. In addition to the high cost and data complexity challenges, the application of metagenomic approaches in food safety is limited by several other factors. First, the standardization of protocols remains a significant issue. Differences in DNA extraction methods, sequencing platforms, and bioinformatic pipelines can result in inconsistent and noncomparable results across laboratories. Second, bioinformatic challenges such as the management of large datasets, the risk of false positives due to low‐quality reads or contamination, and the lack of reference genomes for many environmental microbes' complicate data interpretation. Moreover, biases introduced during DNA amplification or sequencing, such as GC‐content bias or preferential amplification, can distort the true microbial composition. Therefore, validation studies and cross‐methodological comparisons are essential to increase the reliability of metagenomic findings in real‐world food safety applications. Addressing these shortcomings will be key to integrating metagenomics into routine quality control systems (Bianconi et al. 2023; Satam et al. 2023; Oon et al. 2023; Zhao et al. 2025).

In recent years, due to the increase in microbiological food safety problems, the use of more advanced and valid analysis methods has become necessary. In this review, how to use metagenomic analysis methods in the rapid and effective determination of microbiological risks within the scope of food safety and their future importance has been considered.

Metagenomic analyses are predicted to make two important contributions to food safety and scientific literature. While the characteristics of microorganisms in food samples are revealed clearly and completely with the metagenomic analysis approach, it is also possible to create a database with data obtained from studies containing thousands of genes (Ucarlı 2022). When the principles of the methods of metagenomic analyses in the studies conducted in recent years are examined, it is observed that the analyses are generally handled with two different principles (Table 2). In the first principle, the genomic structures (gene–gene segment) of microorganisms that cause problems in food safety are examined. In the second principle, the metabolites produced by microorganisms during their life cycles are determined (Ucarlı 2022; Bal et al. 2022; Cerit 2022; Chen et al. 2022). Metagenomic techniques employed in food safety studies typically fall under three categories (Sadurski et al. 2024; Zhou et al. 2015; Breitwieser et al. 2019; Jagadeesan et al. 2019; Rashid et al. 2025).

1. **16S rRNA gene sequencing**: An amplicon‐based method targeting conserved bacterial regions for taxonomic profiling.
2. **Shotgun metagenomics**: An untargeted sequencing of all genetic content, providing detailed insights into taxonomic composition and functional genes.
3. **Deep metagenomic or high‐throughput sequencing**: Terms often used interchangeably with shotgun methods, referring to advanced sequencing depth and coverage for rare taxa detection.

**TABLE 2:** Recent findings obtained in foods via metagenomic analyses.

Food	Objective	Finding	Method	Principle	References
Kombucha	‐ Investigation of the microbial flora of SCOBY made from telang flower—Evaluation of microorganisms for food safety	Acetic acid bacteria were found to be more dominant than lactic acid bacteria. *Komagataeibacter* was the most common bacteria in Kombucha, while *Dekkera bruxelensis* was the most common yeast. No pathogenic bacteria or heavy metal contaminants were detected in Telang flower kombucha.	NGS (Next‐generation sequencing)	Gen base	(Rimbawan et al. 2024)
Cheese	‐Determining the microbiome of Portuguese cheese made with raw sheep milk and comparing PDO and non‐PDO cheeses	The most common bacterial species in cheeses: ○ *Leuconostoc mesenteroides* ○ *Lactococcus lactis* ○ *Lactobacillus plantarum* ○ *Lacticaseibacillus rhamnosus* ○ *Enterococcus durans* ○ *Lactobacillus coryniformis* PDO cheeses were found to have more bacterial diversity than non‐PDO cheeses.	Shotgun metagenomics analysis	Gen base	(Slamandane et al. 2024)
Peanut	‐Determination the diversity of mold communities in peanuts	*Aspergillus* species were identified, and their effects on aflatoxin production were determined.	NGS	Gen base	(Kodape et al. 2024)
White chesee	‐ Characterizing the lactic acid bacteria (LAB) community in a small‐scale white cheese processing plant	177 LAB species were detected and *Lactobacillus lactis* was the most common in all samples	Shotgun metagenomics analysis	Gen base	(Cerit et al. 2024)
Ready‐to‐eat vegetable salads	‐Detecting bacterial communities in ready‐to‐eat (RTE) vegetable salads sold in Kampala, Uganda	Predominantly *Proteobacteria* (65.34%) were discovered, followed by *Firmicutes* (31.60%) and *Bacteroidota* (0.14%). *Deinococcota* (0.01%) and *Planctomycetota* (0.01%) were found, indicating the presence of contaminants of fecal origin in salads.	16S rRNA	Gen base	(Kawooya et al. 2024)
Chokot drink	‐Investigating the microbial population of Chokot, a rice‐based fermented drink	The most common bacterial species in Chokot: ○ *Bacillus* ○ *Arthrobacter* ○ *Lactobacillus* ○ *Ilyobacter* ○ *Clostridium* ○ *Lactococcus*	16S rRNA	Metabolit base	(Bhattacharjee et al. 2023)
Bieno cheese	‐Investigate the microbiota of ‘Bieno’ cheese, a traditional cheese produced in the Republic of North Macedonia	The dominant species ○ *Streptococcus* ○ *Lactococcus* ○ *Bifidobacterium* ○ *Staphylococcus* ○ *Enterococcus* *Acinetobacter johnsonii* was also dominant spicies.	16S rRNA	Gen base	(Josifovska et al. 2024)
*Cheonggukjang*	‐Determining the microbiota of the naturally fermented Korean fermented soya bean cheonggukjang	*Bacillus thermoamylovorans* was found to be the most abundant species in Cheonggukjang. The other species found in abundance: ○ *Bacillus licheniformis* ○ *Bacillus glycinifermentan*, ○ *Bacillus subtilis* ○ *Bacillus paralicheniformis* ○ *Bacillus amyloliquifaciens* ○ *Brevibacillus borstelensis* ○ *Brevibacillus sonorensis*	Shotgun metagenomics analysis	Metabolit base	(Tamang et al. 2022)
Fermented vegetables	‐Identifying probiotics in pickles commonly consumed in the Middle East, Africa, and the Asian subcontinent	Dominant species detected from samples: ○ *Levilactobacillus namurensis* ○ *Lentilactobacillus buchneri* ○ *Lentilactobacillus parafarraginis* ○ *Lactiplantibacillus pentosus* ○ *Pectobacterium carotovorum* ○ *Leuconostoc carnosum* ○ *Weissella confuse*	Shotgun metagenomics analysis	Gen base	(Yasir et al. 2022)
Yoghurt	‐Determining the microbiota of homemade buffalo yoghurt from five different local producers in Amasra province	One of the yoghurt samples contained *Staphylococcus aureus, two contained Klebsiella pneumonia*, and all contained *Salmonella enterica* . Hygiene rules should be more strictly observed during homemade production, processing, and sale of these products in public markets.	NGS	Gen base	(Delikanlı‐Kiyak and Yılmaz 2024)
Kımız	‐Determining the microbiota of kımız	Four new species belonging to the genera *Lactobacillus, Streptococcus, Acetobacter*, and *Rothia* were described.	Deep metagenomic sequencing	Metabolit base	(You et al. 2022)
Toddy drink	‐ Determining the microbial flora of Toddy, an Indian fermented date drink	Dominant species detected from samples: ○ *Leuconostoc mesenteroides* ○ *Leuconostoc citreum* ○ *Lactobacillus helveticus* ○ *Lactiplantibacillus plantarum* ○ *Lactococcus lactis* ○ *Acetobacter malorum* ○ *Gluconobacter japonicus* ○ *Gluconacetobacter liquefaciens* ○ *Fructobacillus durionis* ○ *Zymomonas mobilis* ○ *Saccharomyces cerevisiae* ○ *Hanseniaspora uvarum* ○ *Hanseniaspora guilliermondii*	Shotgun metagenomics analysis	Metabolit base	(Das and Tamang 2023)
Huangjiu drink	‐ Determination of the flavor and quality formation mechanisms of Chinese rice wine Huangjiu based on its microbial metabolism.	Regional microbial evidence was found related to the geographical characteristics of Huangjiu.	NGS	Gen base	(Luo et al. 2024)
Lettuce	‐ Determining the metagenomic evaluation of lettuce rhizome from Talton, Gauteng Province, South Africa	Dominant species detected from samples: ○ *Acinetobacter* ○ *Pseudomonas* ○ *Streptomyces* ○ *Candidatus* ○ *Solibacter* ○ *Burkholderia* ○ *Bradyrhizobium* ○ *Mycobacterium*	Shotgun metagenomics analysis	Gen base	(Babalola et al. 2023)
Tibetan kefir grains enriched with selenium	‐Determining the microbiological composition of selenium‐enriched Tibetan kefir grains (Se‐TKGs)	The most abundant microbial species detected from samples: ○ *Lactobacillus kefiranofaciens* ○ *Lactobacillus helveticus*	Shotgun metagenomics analysis	Gen base	(Chen et al. 2024)
Kombucha	‐Determining the genetic characterization of kombucha produced with black tea and cocoa bean shell infusion	Microorganisms belonging to the fungi and bacteria kingdoms of *Saccharomycetaceae* and *Acetobacteraceae* families were found.	16S rRNA gene sequencing		(de Oliveira Duarte et al. 2024)
Miso	‐Determining the microbial ecology of miso (a Japanese fermented paste)	The detection of human‐specific *Staphylococcus epidermidis* strains highlights that the risk of human food contamination is more widespread than currently known.	16S RNA	Gen base	(Kothe et al. 2024)
Spinach	‐Benchmarking short‐read assemblers for metagenomic identification of food and waterborne pathogens using simulated bacterial communities.	Multiple drug‐resistant *Pseudomonas aeruginosa* was found in the samples.	Shotgun metagenomics analysis	Gen base	(Chen and Meng 2022)
Kefir	‐Identifying kefir microorganisms	Kefir grains were a more stable community with distinct dominant species than milk kefir.	Shotgun metagenomics analysis	Gen base	(González‐Orozco et al. 2023)
Dry cured fish	‐Identifying microbiological safety of dry cured fish	The most abundant phyla: ○ *Pseudomonadota* ○ *Bacillota* ○ *Mycoplasmatota*	Shotgun metagenomics	Gen base	(Indio et al. 2024)
White cheese	‐Identifying the safety of unpackaged Turkish white cheeses sold in public markets in Ankara	*Staphylococcus aureus*, *E. coli* , and *Salmonella* were found. Poor hygiene and sanitation conditions are found in cheeses.	NGS	Gen base	(Kahraman‐Ilıkkan and Bağdat 2022)
Van Herb Cheese	‐Determining microbial ecology of herb cheese	*Weissella jogaejeotgali* was detected in 15 cheese samples. Including different herbs in the production did not affect the bacterial diversity and microbial composition.	NGS	Gen base	(Rustemoğlu et al. 2023)
Monascus fermented cheese	‐Examining the taxonomic and functional changes in microorganisms in cheese samples in order to clarify the role of microorganisms in flavor formation	The genera identified as related to flavor ○ *Monascus* ○ *Lactococcus* ○ *Aspergillus* ○ *Lactiplantibacillus* ○ *Staphylococcus* ○ *Flavobacterium* ○ *Bacillus* ○ *Clostridium* ○ *Meyerozyma* ○ *Enterobacter*	16S rRNA	Metabolit base	(Wang et al. 2024)
Raw milk	‐Investigating raw milk microbiota and microbiota relationships with climate variables and chemical composition over 12 months in various parts of Ireland.	All samples were found to contain a core microbiota containing the following bacteria: ○ *Pseudomonas* ○ *Lactococcus* ○ *Acinetobacter* ○ *Leuconostoc*	16S rRNA	Metabolit base	(Yap et al. 2024)
Tomatoes	‐Investigating surface microbiota on processed tomatoes from 10 samples from two main production areas in Southern and Northern Xinjiang.	*Alternaria and Fusarium* have been found to be plant pathogenic fungi. Foodborne pathogens such as *Pantoea, Erwinia, Enterobacter, Enterococcus*, and *Serratia* are also included.	16S rRNA	Gen base	(Li et al. 2024)
Sashimi	‐Identifying microbiome composition of sashimi as it is served and ready to eat.	*Salmon sashimi* has the highest microbiome diversity.	16S rRNA	Metabolit base	(Ho et al. 2024)
Dried shrimp	‐Examining the effects of the production process on the microbiota and metabolites in dried shrimp.	*Vibrio, Photobacterium, and Shewanella* were the primary pathogenic bacteria in shrimp samples. *Lactococcus lactis* has been identified as a significant potential beneficial microorganism.	16S rDNA	Metabolit base	(Yu et al. 2024)
Meat	‐Comparing the growth and survival of *Escherichia coli* HEHA16, *Listeria monocytogenes, Salmonella enterica* Typhi, *Cronobacter sakazakii and* a cocktail of these bacteria in sterile broths from ground chicken, pork and beef, as well as pea‐based and soy‐based ground meats.	All flesh‐like fluid provided suitable conditions for the growth and proliferation of the bacteria studied, except for *Escherichia coli* HEHA16. The flesh‐like fluid primarily supported the survival of *Listeria monocytogenes* but, to a lesser extent, also supported the survival of *Cronobacter sakazakii* .	NGS	Gen base	(Bonaldo et al. 2023)
Ezine cheese	‐Identifying the initial bacterial consortium of Ezine PDO cheeses	Dominant species detected from samples: ○ *Streptococcus thermophilus* ○ *Lactobacillus graminis*	NGS	Gen base	(Ozturk et al. 2022)
Brazilian cheeses	‐Identifying the microbiota of five Brazilian artisanal cheese types from Brazi's Southern and Southeastern regions	The most common microorganism is *Lactococcus lactis* subsp. *lactis* Followed in order: ○ *Streptococcus thermophilus*, ○ *Corynebacterium variabile* ○ *Diutina catenulata*, ○ *Debaryomyces hansenii* ○ *Kodamaea ohmeri*	16S/ITS	Gen base	(Kothe et al. 2022)
Traditional fermented foods of Hainan, China	‐Investigating microbial diversity in fermented foods and their effects on food quality and sustainability and understanding how these affects human physiology	*Lactiplantibacillus plantarum* was the dominant species in fermented Yucha and *Lactiplantibacillus* in fermented vegetables. The analysis of *Lactiplantibacillus plantarum* genetic polymorphism would contribute to understanding this strain's evolutionary history.	Shotgun metagenomics analysis	Gen base	(Zhang et al. 2021)
Poultry meal pet food ingredients	Determining those changes in the food microbiome can be used to indicate unexpected contaminants or environmental changes.	The most abundant were: ○ *Bacteroides* ○ *Clostridium* ○ *Lactococcus* ○ *Aeromonas* ○ *Citrobacter* Microbiome sequencing can be used to characterize complex food microbial communities.	RNA sequencing	Metabolit base	(Beck et al. 2021)
Food service facility kitchen surfaces	‐Assessment of spatial and temporal variations in potential cross‐contamination pathways	The most common microorganism on food service facility kitchen surfaces: ○ *Bacillus* ○ *Acinetobacter* ○ *Streptophyta* ○ *Enterobacter* ○ *Pseudomonas* ○ *Serratia* ○ *Enhydrobacter* ○ *Staphylococcus* ○ *Paracoccus* ○ *Lysinibacillus*	16S rRNA	Gen base	(Lim et al. 2021)
Beef, pork, chicken	‐ Determining of the viral metagenomic status of beef, pork and chicken sold in supermarkets in southern Brazil	Two new porcine genomoviruses have been isolated from pigs. Although neither of these viruses has a history of transmission to humans, the findings indicate that such agents are inevitably consumed through the food chain.	Metagenomic sequencing	Gen base	(Cibulski et al. 2021)
Milk	‐Identificating bacterial composition in milk from Korean farms	The proportion of bacteria that can cause spoilage varies from province to province.	16S rRNA	Gen base	(Ryu et al. 2021)
Cheese	‐Systematic characterization of microorganisms found in traditional cheeses on their rinds using cultural, genomic, and metagenomic methods	All genomes sequenced within the study's scope were used as complementary references to those found in databases. Creating co‐occurrences and network analysis systems was provided as the first step of ecosystem reconstruction.	16S rRNA and *rpoB* gene sequence	Gen base	(Kothe et al. 2021)
Cyprus Halloumi	‐Characterizing bacterial diversity in traditional Cyprus Halloumi	The variations detected among the bacterial communities emphasize that the initial microbiome found in raw milk and surviving after pasteurization and the factors associated with Halloumi production conditions contribute to the shaping of the final microbiota composition.	High‐throughput sequencing	Gen base	(Kamilari et al. 2020)
Skimmed Milk Powder	‐Traceability of microorganisms that may pose potential risks to quality and safety	Microbiota of raw milk was very diverse, but psychrotrophic *Pseudomonas* and *Acinetobacter*, which are associated with spoilage, were present in all samples. More specifically, it was emphasized that metagenomic analyses can trace microbial species from raw milk to processing and final powder products.	16S rRNA	Gen base	(Aoife et al. 2020)
Leaf vegetables	‐Determining of human norovirus infection and transmission patterns	Food and water are potential vehicles for transmitting more than one foodborne virus.	NGS	Gen base	(Liu et al. 2020)
Arabian laba	‐Investigating bacterial diversity and function of laba traditionally served in the Middle East, Africa, and the Indian subcontinent	Homemade laba had higher bacterial diversity and higher probiotic bacteria and pathogen content. Antimicrobial resistance genes and transposases were found in homemade laba.	16S rRNA	Gen base	(Yasir et al. 2020)

**FIGURE 4:**
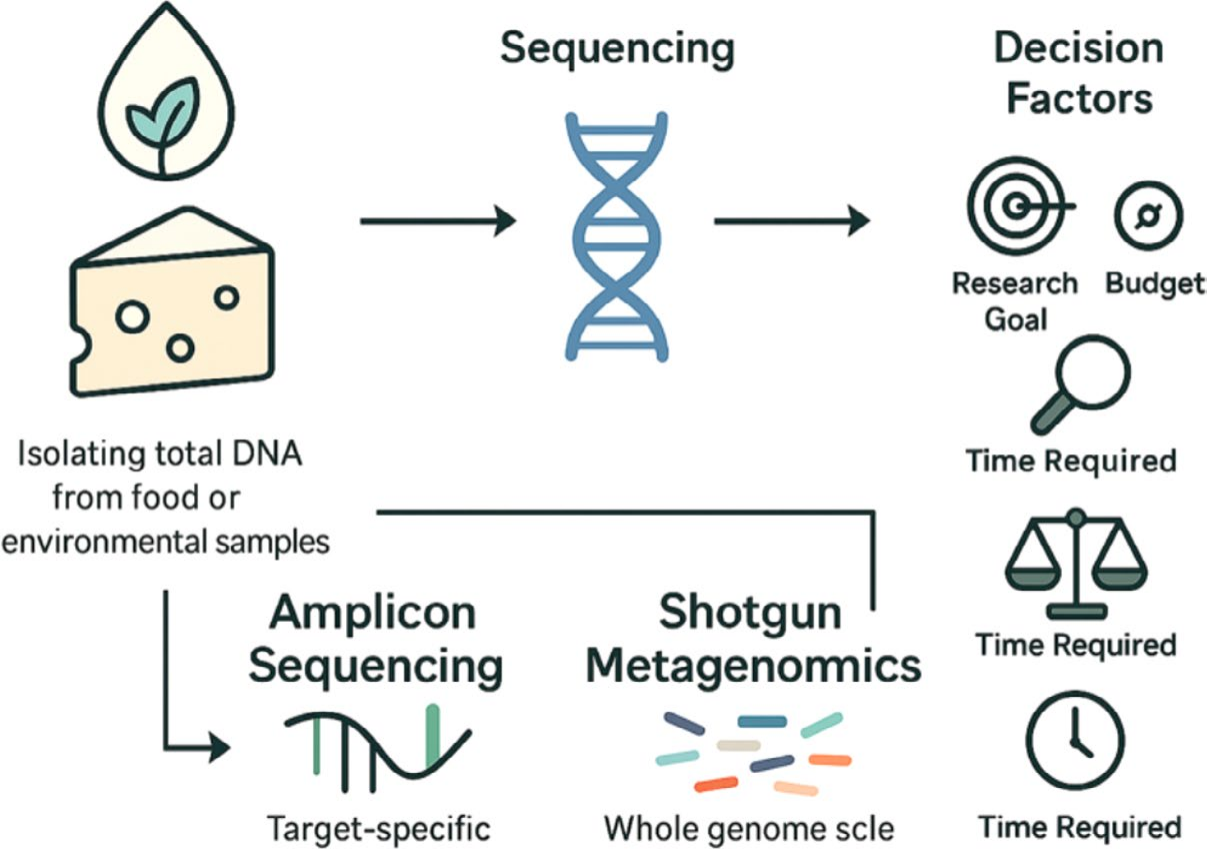
Approaches for metagenomic analysis.

Table 2 summarizes studies across these methods, with an added column indicating the specific technique used for each caseMetagenomic analysis involves the extraction of DNA directly from food or environmental samples, followed by sequencing and bioinformatics‐based taxonomic or functional identification of microorganisms. There are two main approaches: amplicon sequencing, which targets conserved genetic regions such as the 16S rRNA gene for bacterial profiling; and shotgun metagenomics, which sequences all genetic material in a sample, providing deeper taxonomic resolution and insights into microbial functions (Figure 4) (Sadurski et al. 2024; Breitwieser et al. 2019; Zaheer et al. 2018; Aplakidou et al. 2024). Both approaches utilize next‐generation sequencing (NGS) platforms such as Illumina or Oxford Nanopore. The choice of method depends on the research objective, desired resolution, and resource availability (Sadurski et al. 2024; Breitwieser et al. 2019; Zaheer et al. 2018; Aplakidou et al. 2024).

Resource allocation in metagenomic analyses is influenced by multiple factors, including budget constraints, desired taxonomic resolution, and the specific goals of the study. 16S rRNA sequencing, being relatively cost‐effective and less computationally intensive, is often preferred by academic and public health laboratories for broad microbial community profiling. In contrast, shotgun metagenomics requires greater resource investment—both in terms of sequencing depth and bioinformatics infrastructure—but offers superior taxonomic and functional resolution. Industrial stakeholders, such as food manufacturers or commercial testing labs, tend to allocate resources toward targeted NGS platforms that are faster and more scalable, particularly for routine quality control. Research‐intensive institutions and regulatory bodies, on the other hand, are increasingly investing in deep shotgun metagenomics for tasks such as source tracking, antimicrobial resistance profiling, or virome analysis. The trend is gradually shifting toward hybrid approaches and cloud‐based analysis tools, which reduce in‐house resource needs while still enabling high‐resolution metagenomic insights. As sequencing costs continue to decline and bioinformatics becomes more automated, broader adoption across sectors is expected (Sadurski et al. 2024; Imanian et al. 2022).

Amplicon sequencing and shotgun metagenomic testing methods are used to determine microbial diversity (Nam et al. 2023). Both methods have some advantages and disadvantages. Before starting a metagenomic study, the technique to be used, the characteristics of the microbial population to be identified, the budget allocated for the study, and the method to be used in the implementation phase should be decided. For example, while the metagenomic amplicon analysis method is more suitable for identifying lactic acid bacteria in the microbiota of fermented foods, the shotgun metagenomic analysis method is used to identify all microbiota in the sample (Cerit 2022; Hora 2018; EFSA (European Food Safety Authority) 2019).

**FIGURE 5:**
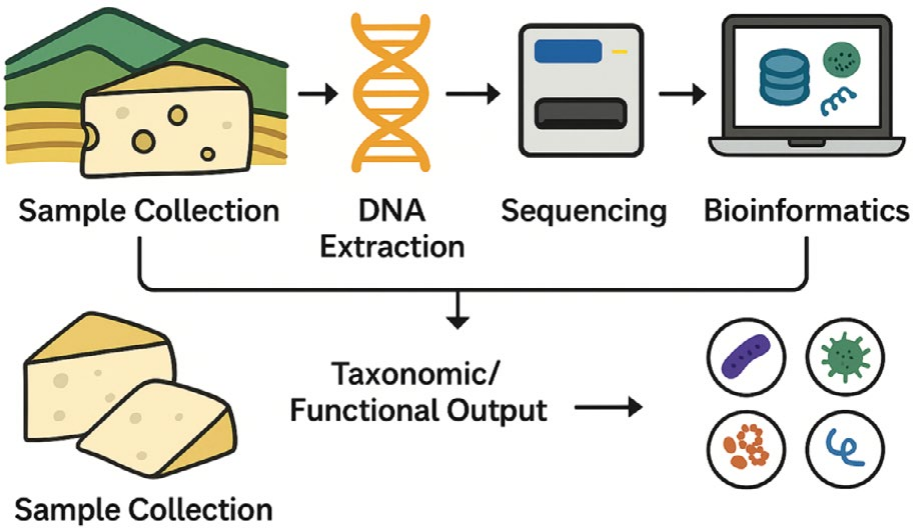
Workflow chart of metagenomic analysis (adopted from (Aktepe and Çakır 2023)).

The primary analysis steps of metagenomic analyses, starting with the sampling phase and continuing with data collection, are shown in Figure 5. Although each process step is important in metagenomic analyses, the most critical step is the reconstruction of individual genes and genomes of microorganisms using computational programs that assemble small sequence DNA fragments generated by metagenomic methods and sequencing tools (ten Hoopen et al. 2017; Lapidus and Korobeynikov 2021; Gao et al. 2021; Liu et al. 2021).

As determined in previous studies, the diversity and content of microorganisms that cause problems in terms of food safety and microbiology vary according to the food product, and in the studies examined, it was observed that the most problematic microorganisms encountered in production were lactic acid bacteria, *Streptococcus, Escherichia coli*, and *Clostridium* species (Table 2). Several studies summarized in Table 2 identified both common and food‐specific microbial species. For instance, *Lactobacillus* species were frequently detected in fermented dairy and meat products, suggesting their role as core microbiota in traditional processing (Cerit et al. 2024; Chen et al. 2024; Slamandane et al. 2024; You et al. 2022; Ozturk et al. 2022). On the other hand, species such as *Pseudomonas* species were unique to minimally processed vegetables, reflecting environmental exposure and storage conditions (Lim et al. 2021; Aoife et al. 2020). The occurrence of unique microbial species in specific food types may be influenced by factors such as processing methods, origin of raw materials, fermentation culture, and hygiene conditions. For example, artisanal cheeses harbored distinct microbial signatures due to region‐specific starter cultures and maturation environments, while seafood samples exhibited unique halophilic bacteria not found in other food types (Indio et al. 2024; Josifovska et al. 2024; Ozturk et al. 2022; Kothe et al. 2022; Kamilari et al. 2020). These findings demonstrate the food matrix‐specific nature of microbial communities, which can be captured effectively through metagenomic analysis offering both taxonomic and ecological insights that would be missed by conventional methods (Walsh et al. 2020; Bloomfield et al. 2025).

The data summarized in Table 2 reveal both core and unique microbial species identified through metagenomic analyses across different food types. For example, in traditional cheeses such as Portuguese PDO variants, *Lactobacillus plantarum* and *Streptococcus thermophilus* were commonly detected, consistent with their known role in fermentation and flavor development. These species were also found in fermented meats, indicating their broad relevance in traditional processing environments (Muñoz‐Martinez et al. 2025; Slamandane et al. 2024).

Conversely, in minimally processed ready‐to‐eat salads, *Pseudomonas* and *Enterobacter* species were prevalent organisms often associated with spoilage and postharvest contamination. These were rarely detected in dairy products, highlighting the impact of processing and storage conditions on microbial diversity (Babalola et al. 2023; de Oliveira Duarte et al. 2024; Kothe et al. 2024; Chen and Meng 2022; Li et al. 2024).

In contrast, unique species such as *Tetragenococcus halophilus* were found only in high‐salt fermented fish, likely due to their halotolerance and metabolic adaptations. Such findings underscore the ability of metagenomics not only to characterize microbial communities but also to infer ecological relationships between food environments and resident microbiota (Rodpai et al. 2021).

A notable example is the detection of coinfections of human norovirus in a foodborne outbreak, where metagenomic fingerprinting enabled source attribution. Although the exact food vehicle was not isolated, the study by Liu et al. (Liu et al. 2020) demonstrated the value of metagenomics in outbreak investigation and viral pathogen detection.

In‐depth examination of the microbiome, especially in fermented food products, with metagenomic analyses provides significant advantages in terms of improving food quality and sustainability. It has been observed that most of the metagenomic studies conducted in recent years have focused on examining the microbiome of fermented products (Cerit et al. 2024; González‐Orozco et al. 2023; Rimbawan et al. 2024; Slamandane et al. 2024).

Several studies presented in Table 2 demonstrate the utility of metagenomic analyses in evaluating the microbial ecology of fermented foods. For example, in traditional cheeses, metagenomic sequencing revealed the presence of core lactic acid bacteria such as *Lactobacillus plantarum* , *Leuconostoc mesenteroides* , and *Streptococcus thermophilus* , which play essential roles in acidification, flavor development, and preservation (Das and Tamang 2023; Slamandane et al. 2024; Ozturk et al. 2022; Kothe et al. 2022). Moreover, shotgun metagenomics enabled the detection of functional genes related to proteolysis, lipolysis, and even bacteriocin production, shedding light on the biochemical processes during fermentation. The ability to identify both dominant and subdominant microbial players offers a more complete understanding of fermentation dynamics compared to traditional methods (Ercolini 2013). These findings support the use of metagenomics not only in safety monitoring but also in quality optimization and authentication of fermented products, aligning with the conclusions presented in this review (Srinivas et al. 2022; Kumar et al. 2025).

## 2 Future Directions in Food Safety Metagenomics

The future of metagenomic analyses in food safety lies in their integration with emerging technologies and methodologies. One promising direction is the combination of metagenomics with other “omics” technologies—such as metabolomics, proteomics, and transcriptomics—to provide a multi‐layered understanding of food microbiomes and their functional impacts. These integrated approaches can enhance pathogen detection, microbial ecology assessments, and functional predictions (Coppola et al. 2025; Shukla et al. 2025).

Another key area is the use of artificial intelligence (AI) and machine learning (ML) in bioinformatics pipelines. AI can automate large‐scale sequence data processing, reduce noise, and predict contamination risks based on complex patterns across datasets. These technologies also hold promise for real‐time decision‐making in food quality control (Roy et al. 2024). Additionally, the development of low‐cost, high‐throughput sequencing platforms, such as nanopore and microfluidic‐based devices, will make metagenomic analyses more accessible to small‐ and medium‐sized food enterprises (Latorre‐Pérez et al. 2020; Kaur et al. 2022; Herbert et al. 2025). Future research should also address ethical concerns, particularly in relation to data sharing, privacy, and the use of microbial data from traditional and local food systems. Establishing standardized databases and protocols under global regulatory frameworks (e.g., FAO, WHO, EFSA) will be essential for harmonizing metagenomics‐based food safety systems (Singh and Kumar 2025).

Overall, the next decade will likely witness the integration of interdisciplinary technologies in food metagenomics, leading to more accurate, cost‐effective, and globally applicable safety systems (Sadurski et al. 2024; Imanian et al. 2022; Ko et al. 2022).

## 3 Emerging Trends and Regulatory Considerations

In recent years, metagenomics has not only revolutionized food safety but also started to influence broader areas such as personalized nutrition and sustainable food systems. By characterizing individual‐specific microbiota in food consumption patterns, metagenomic tools offer valuable insights into personalized dietary interventions. Similarly, the ability to monitor microbial shifts during sustainable food production processes aligns metagenomics with environmental goals and circular bioeconomy strategies (Sadurski et al. 2024; Muñoz‐Martinez et al. 2025; Coppola et al. 2025).

From a regulatory standpoint, however, the routine implementation of metagenomic analysis still faces gaps. There is a need for harmonized standards, legal frameworks, and quality assurance protocols to ensure the validity and reliability of metagenomic data, particularly in foodborne outbreak investigations. Institutions such as the EFSA, FDA, and Codex Alimentarius have initiated working groups to address these gaps and propose guidelines for the safe and effective use of genomics in food monitoring systems (Codex Alimentarius Commission 2023; EFSA BIOHAZ Panel 2019; Food and Drug Administration (FDA) 2024).

## 4 Conclusions

In recent years, it has been observed that scientists have focused on molecular techniques based on genetic material and traditional culturing methods for the identification of microorganisms. In addition to culture‐based molecular methods for environmental samples, the application of metagenomic technologies has made it possible to sequence the genome of the microbial load and identify microorganisms, bringing a new perspective to microbiota studies. The great majority of recent studies have adopted metagenomic approaches instead of traditional culture‐based methods, thus reflecting a paradigm shift in food safety microbiology. Metagenomics can provide fast and effective solutions to the healthy nutrition problems of consumer societies, whose expectations and awareness are rapidly increasing. In the process covering all stages from farm to table in the production of food products, microbial contamination can occur from many different sources. In addition, the identification of microbiota in food or the determination of which microorganisms produce the products formed during fermentation and at which stage can be revealed by metagenomic analyses. In short, metagenomics is accepted as an auxiliary discipline in terms of food microbiology and food safety. In this way, which strains are involved in foodborne poisoning and the resistance factors affecting the development of these microorganisms, microbial loads at critical control points in the production line, and microbial contamination sources, and microorganisms effective in fermented food production can be determined quickly and effectively.

In the near future, metagenomic analyses will inevitably become widespread for the rapid and highly accurate identification of genes and/or protein metabolites in foods and microorganisms that cannot be produced by the traditional petri plate method and for meeting the necessary quality and food safety criteria.

## Author Contributions

**Berrak Delikanli‐Kiyak:** conceptualization (equal), investigation (equal), methodology (equal), resources (equal), supervision (equal), validation (equal), writing – original draft (equal), writing – review and editing (equal). **Ilkay Yilmaz:** conceptualization (equal), investigation (equal), methodology (equal), resources (equal), supervision (equal), validation (equal), writing – original draft (equal), writing – review and editing (equal). **Metın Guldas:** conceptualization (equal), investigation (equal), methodology (equal), resources (equal), supervision (equal), validation (equal), writing – original draft (equal), writing – review and editing (equal).

## Disclosure

Generative AI Declaration: Figures 1 through 5 in this revised version were generated using generative AI‐based design tools (such as ChatGPT image module) to enhance visual clarity and academic quality in response to reviewer feedback. The conceptual content, layout, and scientific descriptions of these figures were entirely designed and controlled by the authors. No generative AI tools were used in the writing of the manuscript or in data collection and analysis. The authors take full responsibility for the accuracy, integrity, and interpretation of all AI‐assisted visual elements.

## Conflicts of Interest

The authors declare no conflicts of interest.

## Data Availability

This review article is based on previously published studies, all of which are cited accordingly. No new data were generated, and therefore, data sharing is not applicable.
